# The Causes and Prevention of Trench Foot

**Published:** 1918-02

**Authors:** 


***The Causes and Prevention of Trench Foot.*** By BASIL HUGHES, Capt., R. A. M. C. (T. F.). Abs. from the *Brit. Med. Jour.*, May 20, 1916.

The author, in seeking to discover the cause of trench feet, was obliged to give up a belief he was tempted to hold that the condition was due to infection from organisms present in the mud. When rubber shoes were provided for the men, there was no appreciable drop in the incidence. To overcome the effects of fatigue and long exposure, every effort was made to supply the men with the most appetizing and nourishing food. This effort combined with the supply of rubber boots prevented advanced stages of the malady, but the incidence of the early stage, that is, the period of initial edema, of pains, and inflammation, was still disappointingly high.

H. noticed on one occasion when he sat for a moment on the firing step to rest, that his feet quickly " went to sleep ". He repeated this experience on two other occasions, and found that each time the same effect was produced. This led him to suppose that the sitting posture usually maintained by the men on the firing step during rest periods, was an important contributory cause to the condition of trench feet. The result of this posture is to create pressure upon the popliteal space, thus impeding the return of blood along the popliteal artery. Often the men go to sleep in this position.

The author concluded that the condition of trench feet is caused by two factors :

1. A predisposing factor, namely, fatigue.

2. An exciting factor which is purely mechanical, namely, venous stagnation and consequent exudation of material into the tissues of the foot.

The sitting posture on the firing step becomes a serious predisposing condition. The men should be instructed to take their rest by reclining with their feet well up on the firing step wrapped in blankets. The author also believes that hot soup and rum rations are valuable assets in the trenches. Change of socks, even with rubber boots, is also necessary. In addition, the author has found massage given in a special dugout to men with a tendency to trench feet, to be helpful in keeping his squad free from the malady.

